# Lateralizing value of interictal epileptiform discharges and other parameters in hypothalamic hamartoma

**DOI:** 10.1111/epi.18217

**Published:** 2025-01-15

**Authors:** Friederike Niedermoser, Sarah M. Metzger, Kathrin Wagner, Peter C. Reinacher, Jan Schönberger, Julia Jacobs‐LeVan, Andreas Schulze‐Bonhage, Kerstin Alexandra Klotz

**Affiliations:** ^1^ Department of Neuropediatrics and Muscular Disorders, Medical Center, Faculty of Medicine University of Freiburg University of Freiburg Freiburg im Breisgau Germany; ^2^ Epilepsy Center, Medical Center, Faculty of Medicine University of Freiburg University of Freiburg Freiburg im Breisgau Germany; ^3^ Department of Stereotactic and Functional Neurosurgery, Medical Center, Faculty of Medicine University of Freiburg University of Freiburg Freiburg im Breisgau Germany; ^4^ Fraunhofer Institute for Laser Technology Aachen Germany; ^5^ Hotchkiss Brain Institute and Alberta Children's Hospital Research Institute, University of Calgary Calgary Alberta Canada; ^6^ Department of Neuropediatrics University Hospital Bonn Bonn Germany

**Keywords:** bilateral, biomarker, interictal epileptiform discharges, semiology, stereotactic radiofrequency thermocoagulation

## Abstract

**Objective:**

Hypothalamic hamartomas (HHs) are associated with pharmacoresistant epilepsy. Stereotactic radiofrequency thermocoagulation (SRT) shows promise as a disconnecting intervention. Although magnetic resonance imaging (MRI) is typically used to determine the attachment and intervention side, it presents challenges in cases of bilaterally attached HH, where the epileptogenic side is unclear. The lateralizing potential of electroclinical parameters in such cases remains uncertain. This retrospective study evaluates the lateralization value of specific parameters, particularly in patients with unilateral HH, to improve future diagnostics and treatment approaches for bilateral HH.

**Methods:**

Four lateralizing parameters—semiology, ictal electroencephalography (EEG), and interictal epileptiform discharges during awake (IEDs_***w***_) and sleep states (IEDs_***s***_)—were assessed for correlation with HH attachment side using Spearman's ρ. We calculated areas under the curves (AUCs) and cutoffs for left and right IED_***s***_ prognostic lateralizing value, plotting differences between IED_***s*** right_ and IED_***s*** left_ in a receiver‐operating characteristic(ROC) curve to establish the required preponderance of unilateral IEDs_***s***_ to differentiate between left and right HHs. Binomial logistic regression was employed to predict the HH attachment side.

**Results:**

We included 25 patients (2–55 years of age) with mainly unilateral (*n* = 22) HHs who underwent SRT and presurgical evaluation. All parameters correlated with HH attachment side (semiology *R* = −.62, *p* = .005; ictal EEG *R* = .51, *p* = .047; IED_***s***_ *R* = .55, *p* = .018; IED_*w*_, *R* = .61, *p* = .018). AUC values for right and left IED_***s***_ were .76 (*p* = .047) and .85 (*p* = .019), respectively, with cutoffs of .34 and .15. The AUC for “IED_***s*** right_–IED_***s*** left_” was .98 (*p* = .0018) with a cutoff of .16. IEDs_***s***_ and semiology were significant predictors, achieving 88% correct lateralization.

**Significance:**

IEDs_***s***_ are promising biomarkers for HH lateralization in unilateral HH. The predominance of unilateral IEDs_***s***_ suggests ipsilateral HH. Even in cases with predominantly bilateral IEDs_***s***_, a slight preponderance of unilateral IEDs_***s***_ can indicate the attachment side. In addition, combining IEDs_***s***_ and semiology provides a predictive model for HH lateralization.

**Key points**

- As a proof‐of‐concept, we found in 25 patients with mainly unilateral hypothalamic hamartomas (HHs), certain pre‐interventional electroclinical and semiological markers provide lateralizing value beyond magnetic resonance imaging (MRI).
- Interictal epileptiform discharges (IEDs) during sleep and lateralizing seizure semiology emerged as particularly effective predictors for identifying the attachment side of HHs.
- HH seizure semiology offers lateralizing value, often with an asymmetric smile (gelastic) or followed by unilateral tonic facial/extremity movements.
- Over half of seizures, especially gelastic ones, lack recognizable patterns on scalp electroencephalography (EEG), making it unreliable for lateralizing the primary epileptogenic network.
- Incorporating these predictors can aid in selecting the optimal stereotactic radiofrequency thermocoagulation (SRT) intervention side for patients with bilateral HH when MRI alone is not sufficient.

## 1 INTRODUCTION

Hypothalamic hamartoma (HH) is a rare congenital lesion originating from the base of the third ventricle, tuber cinereum, or mammillary bodies, often causing drug‐resistant epilepsy and epileptic encephalopathy.^1^ Gelastic seizures are a characteristic and often early seizure type, with up to 75% of patients also experiencing additional seizure types including focal and focal to bilateral tonic–clonic seizures.^2^ Seizures typically worsen in frequency and severity with disease duration.^3^ Many patients with HHs also exhibit severe comorbidities such as pubertas praecox, psychiatric, developmental, and behavioral disorders.^4^

Given the pharmacoresistant nature of HH‐induced seizures, surgical intervention is usually necessary for seizure control.^5^ Various surgical approaches have been employed, depending on HH location, size, and available expertise.^6^ Laser interstitial thermal therapy (LITT) is used increasingly for smaller HHs, offering real‐time monitoring via magnetic resonance imaging (MRI) thermometry.^7^ Stereotactic radiofrequency thermocoagulation (SRT) is another minimally invasive, ablative technique with recent strategies focusing on minimizing coagulation sites while ensuring complete disconnection at the hypothalamus border.^8^, ^9^, ^10^ In the largest study to date, the overall seizure‐freedom rate after one or repeated SRT was 73%, with a median follow‐up time of 61 months.^11^ Accurate targeting is essential for effective treatment. Shirozu et al. demonstrated that electrophysiological and MRI‐determined borders align in unilateral HH.^12^ However, ~35% of HHs appear bilaterally attached on MRI, complicating ablation side.^1^ Data from ictal single‐photon emission computerized tomography (SPECT) suggest that even in cases where bilateral HHs do not exhibit distinct anatomic lateralization in MRI, they demonstrate functional lateralization.^13^, ^14^ Although bilateral HHs have been treated successfully with SRT, the role of electroclinical information in guiding optimal disconnection remains unclear.^14^ A recent approach proposes coagulating both sides simultaneously; however, an initial unilateral approach is preferred to minimize complications.^15^

In other focal epilepsies, utilization of electrophysiological and semiological data for determining the lateralization of the epileptogenic zone is well‐established, and combining parameters has proven more effective than relying on a single one.^16^, ^17^, ^18^, ^19^ To our knowledge, there are no published data systematically investigate these parameters concerning HH lateralization. Thus our study aimed to investigate four pre‐interventional parameters—seizure semiology, ictal electroencephalography (EEG), and interictal epileptic discharges (IEDs) during wakefulness and sleep—to predict the HH attachment side. We examined patients with unilateral HHs as a proof‐of‐concept and a small group of patients with bilateral HHs where a predominant functional side was assumed.

## 2 MATERIALS AND METHODS

### 2.1 Patients

This retrospective study included patients diagnosed with structural epilepsy due to HHs confirmed by MRI, who underwent at least one SRT at our center between July 10, 2015 and October 31, 2020, with pre‐ and post‐interventional scalp EEG recordings available. The rationale for selecting patients who underwent SRT was based on the hypothesis that disconnection at the hypothalamus border would allow us to infer the dominant side of network involvement in bilateral HHs based on post‐surgical outcomes. Patient records provided demographic, clinical, and surgical data, including age, sex, HH classification, seizure type/frequency, comorbidities, previous treatments, number of SRTs, approach side, seizure outcome, and surgical complications. Complications were classified as acute (improving within 48 h), transient (resolved by 3 months), or persistent (present at last follow‐up). Seizure outcomes were categorized using International League Against Epilepsy (ILAE) and Engel classifications.^20^, ^21^

### 2.2 MRI

HHs were evaluated visually for lateralization by assessing asymmetries and displacements of surrounding structures (fornices, corpora mamillaria, tractus optici, and tractus mamillothalamici) if lateralization was not readily apparent. If lateralization remained undeterminable with the described approach, the HH was considered bilaterally attached. In such cases, the approach side of the first SRT was deemed the dominant functional side if patients were seizure‐free after at least 24 months of follow‐up. HH volume was assessed using multiplanar T2 or T1 reconstruction. Two independent measurements were conducted to determine the maximal extent in all corresponding planes (coronal, axial, and sagittal) with mean values calculated. HH visualization and measurements were performed using IMPAX EE R20 (AGFA HealthCare). Delalande classification was assigned based on T2‐weighted coronary, axial, or sagittal T2 weighting.

### 2.3 EEG acquisition

All patients underwent a minimum 24‐h video‐EEG (VEEG) monitoring prior to SRT. EEG was recorded using the International 10–20 electrode system.^22^ The recording was conducted using Profusion EEG (Compumedics Limited, Abbotsford, Australia) and Natus NeuroWorks EEG software.

### 2.4 EEG rating

In the first step, EEG analysis was independently performed visually by two raters, with 2 and 15 years of EEG analysis experience, respectively. Both raters were blinded to MRI lateralization and surgical intervention side. The event classifications of both raters were compared, and in cases of discrepancies, a consensus classification of interictal and ictal EEG activity was reached in a second step. All EEG recordings were evaluated using both longitudinal bipolar montage (bipolar longitudinal series) and referential montage (common average without Fp), with a low‐pass filter of 70 Hz and a high‐pass filter of .5 Hz applied.

Before any following steps, the entire EEG study was read by one of the raters to select the most representative interictal and ictal segments considering comparable states of vigilance, minimizing artifacts, and ensuring sufficient distance from seizure events before and after. For ictal EEG, the analysis proceeded as follows: (1) 10 consecutive seizures of each seizure type per patient were marked; (2) seizure onset on EEG was localized and assigned to one of four categories: left, right, bilateral, or not identifiable, for example, in the absence of a seizure pattern; and (3) it was verified whether EEG seizure onset occurred before or after clinical seizure onset. Finally, for each patient, the number of EEG seizure onsets assigned to each hemisphere was calculated.

Regarding interictal EEG, the process was as follows. (1) Two intervals during sleep (sleep stage 2 with sleep spindles as an indicator) and two intervals during the awake state, each lasting 5 min with minimal artifacts, were identified. The minimum time between the chosen interictal interval and preceding or subsequent seizures was 2 h whenever possible. If fewer than five IEDs were present in one interval, the period was extended to 10 min, and if still fewer than five IEDs were present, the IED were considered insufficient for classification. (2) All IEDs in all four intervals were marked. (3) IEDs were localized and assigned to one of three categories: independent left, independent right, or bilateral synchronous. This determination was made by identifying the channel where the IEDs appeared first in temporal sequence. In cases of simultaneity, the phase reversal and maximum amplitude of IEDs were considered. Subsequently, the relative frequency of each hemisphere affected was calculated for each patient.

### 2.5 Semiology

VEEG studies were analyzed for early clinical signs of lateralization during seizures. The evaluated period encompassed 5 s before EEG seizure onset until the appearance of the first clinical sign of the seizure. Observations were made regarding the occurrence of these signs and, if present, their attributable hemisphere. Clinical signs were categorized as follows: Ipsilateral signs included automatisms.^23^ Contralateral signs encompassed unilateral somatosensory seizures^24^; asymmetric gelastic seizures^25^; myoclonic, clonic, and tonic activity^26^; dystonic posturing^27^; and versive eye or head turning.^28^ Subsequently, the relative frequency of involvement of each side was calculated for each patient.

### 2.6 Statistical analysis

Statistical analysis was conducted using GraphPad Prism (GraphPad Software, Inc., version 9.3.0). Continuous data were assessed for normal distribution using the D'Agostino‐Pearson normality test and analyzed using either the parametric Fisher's exact test or the nonparametric Mann–Whitney *U* test. The correlation between pre‐interventional parameters and the approach side of the first SRT, as well as the correlation between age at the first SRT and outcome, were calculated using the nonparametric Spearman's rank correlation coefficient. For the first correlation analysis, we pseudo‐ordinally scaled the variables, assigning values of −1 for “left,” 0 for “bilateral,” and + 1 for “right.” Effect sizes were interpreted according to Cohen's correlation scale.^29^ To evaluate the prognostic value of IEDs during awake and sleep states for lateralization, receiver‐operating characteristic (ROC) curves were generated and the area under the curve (AUC) was calculated. Initially this analysis was conducted separately for the right and left sides to demonstrate that the determined cutoff values for each side can effectively determine the probable attachment side of HH in patients with distinct IED distributions. In addition, the difference between IEDs occurring on the right and left sides during sleep was plotted in an ROC curve, to determine the required preponderance of unilateral IEDs during sleep to differentiate between left and right HHs. Only EEG data recorded during sleep were used for the final analysis. Daytime data were excluded due to a very low IED rate that did not allow for robust statistical analysis. The best cutoff values for predicting HH lateralization were determined using the Youden index. A binomial logistic regression was performed to investigate the prediction of HH lateralization based on pre‐interventional parameters. The significance level was set at *α* = .05 for all statistical analyses, with FDR‐adjusted *p*‐values applied where appropriate.

Two hypotheses were tested:

1. The parameters ictal EEG, interictal EEG, and clinical lateralization signs reflect the attachment side of the HHs.
2. A combined analysis of the above parameters is superior to scoring a single parameter alone in determining the HH attachment side.

## 3 RESULTS

### 3.1 Study population

Between August 2010 and January 2021, a total of 26 patients with HHs underwent SRT, and pre‐interventional VEEG monitoring was available. One patient was excluded due to having only intracranial EEG. The cohort comprised 8 adults and 17 children. In 22 patients, the HH attachment side was determined as either right or left based on MRI. In three patients, the HHs were classified as bilateral. Because the predictive value of lateralizing information was not clinically established for HH patients prior to this study, the intervention side for these three patients was selected somewhat randomly. None of the parameters included in this study (e.g., seizure semiology, interictal, or ictal EEG) influenced this decision. All three patients with bilateral HHs (4, 6, and 9 years of age) experienced daily seizures of various types and achieved seizure freedom after a single SRT on the right side in all cases (with follow‐up periods of 48, 63, and 70 months). Detailed patient characteristics are presented in Table 1.

**TABLE 1:** Pre‐interventional patient characteristics (*n* = 25).

Characteristics	Data
Female sex	9 (36)
Patients	
Children	15 (60)
Adults	8 (40)
Age (y) at seizure onset	2.9 ± 3.9
Age (y) at time of VEEG recording	15.52 ± 15.3
Duration (y) of epilepsy	12.64 ± 13.2
Self‐reported seizure types	
Gelastic	19 (76)
Other focal seizures without generalization	21 (84)
Focal to bilateral tonic–clonic seizures	10 (40)
Previous epilepsy surgery	8 (32)
Seed implantation	5 (20.8)
Open or endoscopic resection	3 (12)
Number of different seizure types per patient	2.4 ± .9
Current ASM number	2.2 ± 1.0
Most frequently used ASM	
Oxcarbazepine	13 (23.6)
Levetiracetam	9 (16.3)
Lamotrigine	8 (14.5)
Lacosamide	6 (10.9)
Discontinued ASM	3.7 ± 3.7
Comorbidities	14 (56)
Pubertas praecox	6 (43)
Psychiatric disorders	9 (64)
PTEN Hamartoma tumor syndrome	1 (7)
Hypothalamic disorder	3 (21)
MRI findings	
Volume (mL) HH	3.10 ± 5.01
<1 mL	11 (44)
1–5 mL	9 (36)
>5 mL	5 (20)
Attachment left	8 (32)
Attachment right	14 (56)
Attachment bilateral	3 (12)
Delalande Type 1	0 (0)
Delalande Type 2	14 (56)
Delalande Type 3	8 (32)
Delalande Type 4	3 (12)

*Note*: Data are presented as *n* (%) or mean ± standard deviation.

Abbreviations: ASM, anti‐seizure medication; HH, hypothalamic hamartoma mL; milliliters; y, years.

### 3.2 Ictal EEG and seizure semiology

A total of 25 VEEGs were recorded, with one prematurely terminated due to patient noncompliance. Seizures were detected in all VEEGs. Among the 241 evaluated seizures, 142 (58.9%) were classified as gelastic, 94 (39%) as purely focal‐non‐gelastic, and 5 (2.1%) as focal to bilateral tonic–clonic. Gelastic seizures manifested as mirthless laughter in 64.1% of cases, although awareness could not be reliably assessed in most instances due to the patient's age or the brief duration of the seizures. In younger children, some gelastic seizures progressed to crying behavior; however, these were still classified as gelastic rather than dacrystic based on the first prominent clinical sign. In 26.7% of cases, gelastic seizures were accompanied by an asymmetric smile or tonicity of the mouth, and in 3.5%, they were followed by unilateral tonic movements of the extremities. In 5.6% of cases, the presence of additional motor signs could not be assessed. In 127 seizures (52.7%), no specific seizure pattern was evident on EEG; however, 21 patients (84%) showed at least one seizure type with a discernible pattern. Although four patients (16%) showed no seizure pattern in any type. Three of these displayed lateralizing semiology right after clinical onset in at least one seizure. Early lateralizing seizure semiology on video monitoring was observed in 16 patients (64%) but was present in only 66 (27.4%) of all recorded seizures. Lateralizing signs were present in 44 (46.8%) of gelastic seizures, including asymmetric mouth contractions (40, 91%) and asymmetric clonic or myoclonic hand contractions (4, 9%). Other seizure types showing lateralizing semiology included focal impaired awareness motor onset seizures (10 seizures, 35.6%) with head version (2, 20%) or motor phenomena of the extremities (8, 80%), and focal aware motor onset seizures (10 seizures, 45.5%) with head version (1, 10%) and clonic or myoclonic contractions of the extremities (9, 90%), one bilateral tonic–clonic seizure and one unclassified seizure. Focal aware nonmotor onset seizures did not show lateralizing semiology.

### 3.3 Interictal EEG

Overall, IEDs were present on 24 (96%) of EEG studies during sleep and 19 (76%) during wakefulness. In detail, during wakefulness (during sleep), 8 (3) patients exhibited only right‐sided IED, 1 (0) had only left‐sided IEDs, 4 (8) presented with both right‐ and left‐sided IEDs, 2 (2) showed right‐sided and bilateral synchronous IEDs, and 0 (11) exhibited independent right‐ and left‐sided as well as bilateral synchronous IEDs; 5 (1) had IEDs insufficient for classification. The mean (SD) rate of IEDs during sleep was .19/s (.27).

### 3.4 SRTs and outcome

In this study, a total of 34 SRT procedures were performed in 25 patients, with 8 patients (32%) undergoing more than one procedure. Younger patients at their first SRT tended to experience better outcomes (*R* = .4484, 95% confidence interval [CI] = .05238–.7225; *p* = .047). The size of the HH was not linked to any specific seizure type, as gelastic seizures were present across all subgroups. In addition, there was no significant difference in median HH volume between patients who experienced substantial benefit from SRT (Engel class I) and those who showed little to no improvement (Engel class II–IV) (*p* = .453). All patients with bilateral HHs were seizure‐free after the first SRT intervention. (Please refer to Table 2 for details on interventions and outcomes.)

**TABLE 2:** Overview of interventions, complications, and outcomes in the patient cohort.

Characteristics	Data
Follow‐up period (m)	31 (3–84)
Age at surgery (y)	9 (2–55)
Number of SRT per patient	
1	17 (68)
2	7 (28)
3	1 (4)
Final seizure outcomes (Engel)	
Engel I	
A	12 (48)
B	6 (24)
C	0 (0)
D	0 (0)
Engel II	
A	0 (0)
B	1 (4)
C	0 (0)
D	3 (12)
Engel III	
Ad	0 (0)
B	1 (4)
Engel IV	
A	2 (8)
B	0 (0)
C	0 (0)
Final seizure outcome (ILAE)	
ILAE class 1	11 (44)
ILAE class 2	3 (12)
LAE class 3	2 (8)
ILAE class 4	7 (28)
ILAE class 5	2 (8)
Complications^a^ over all SRT	22 (65)
Acute	8 (30)
Transient	10 (40)
Persistent^b^	3 (12)
Attachment/approach side SRT	
Right/right	14
Left/left	8
Bilateral/right	3
Bilateral/left	0

*Note:* Data are presented as *n* (%), or median (range).

Abbreviations: m, months; y, years.

^a^ Hemiparesis, Horner syndrome, facial paralysis, ptosis, secondary hypothyreosis, diabetes insipidus, fever, nausea, and self‐reported short‐term memory loss.

^b^ Secondary hypothyreosis *n* = 1, mild facial paralysis *n* = 2.

### 3.5 Correlation

**FIGURE 1:**
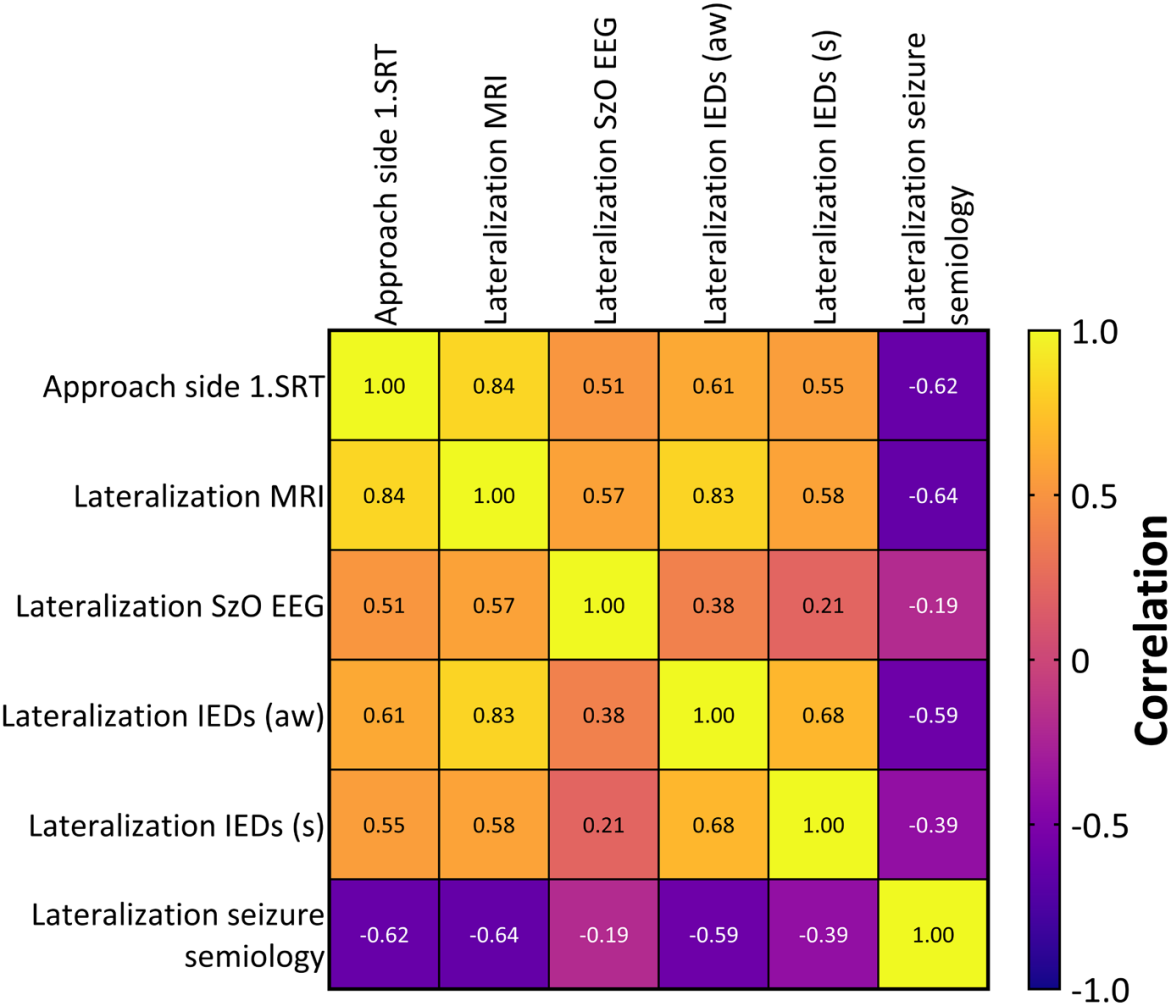
Spearman's correlation matrix showing correlations between the first SRT approach side and the lateralization parameters. aw, awake state; s, sleep; SRT, ?; SzO, seizure‐onset side. Stereotactic radiofrequency thermocoagulation; MRI, Magnetic Resonance Imaging; IED, interictal epileptiform activity; EEG, electroencephalogram

The correlation analysis revealed moderate to strong positive and negative correlations between the approach side of the first SRT and the lateralization of IEDs during wakefulness (*R* = .61, *p* = .018) and sleep (*R* = .55, *p* = .018), as well as the lateralization of EEG seizure‐onset side (*R* = .51, *p* = .047) and the lateralization of seizure semiology (*R* = −.62, *p* = .005) (Figure 1).

### 3.6 ROC curves

**FIGURE 2:**
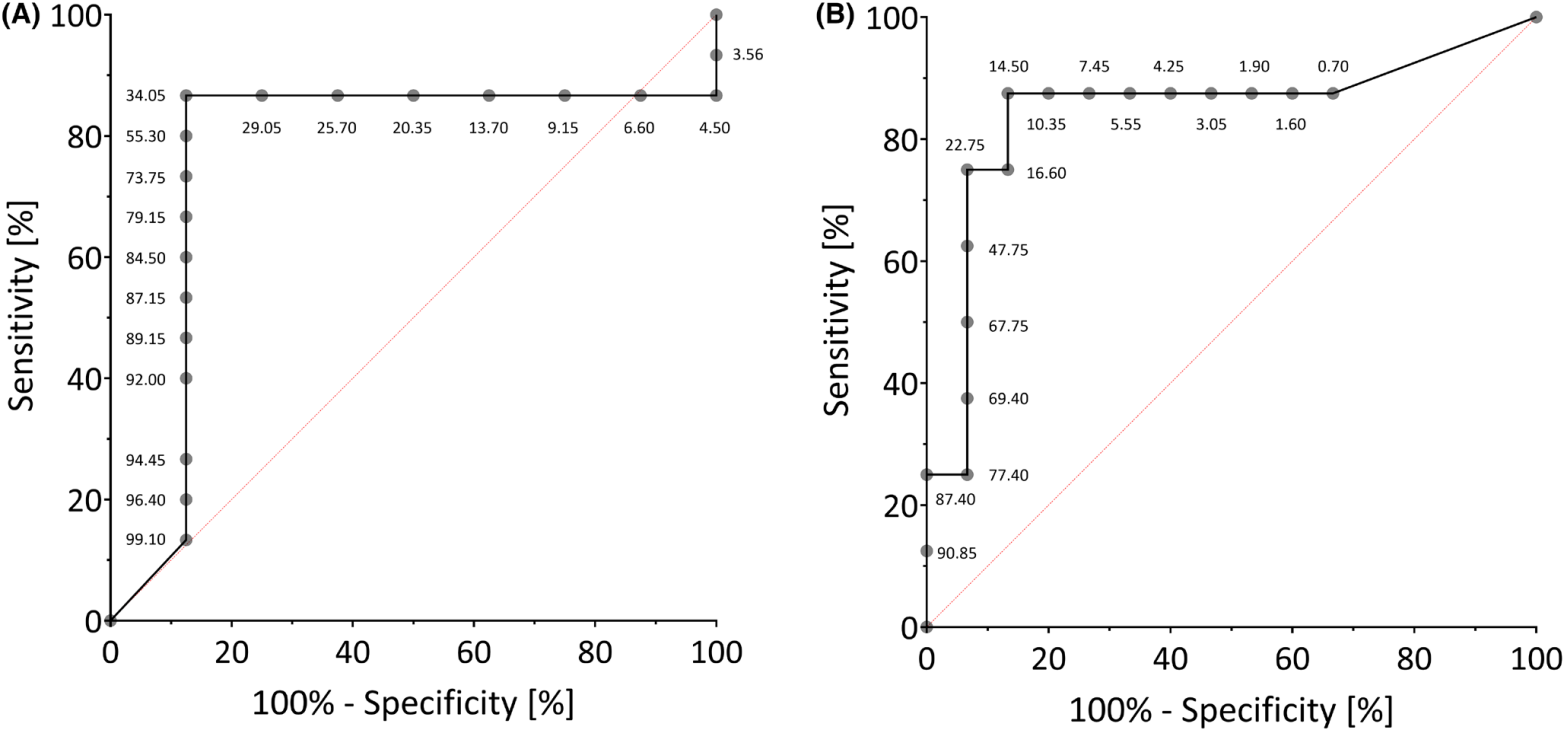
ROC curve of right‐sided IEDs (A) and left‐sided IEDs (B) during sleep. The value pairs sensitivity and 100% specificity for each possible cutoff (IED share on the right/left) are plotted and connected by the black line to form an ROC curve. The red bisector line represents an uninformative ROC curve (sensitivity = 1‐specificity).

**FIGURE 3:**
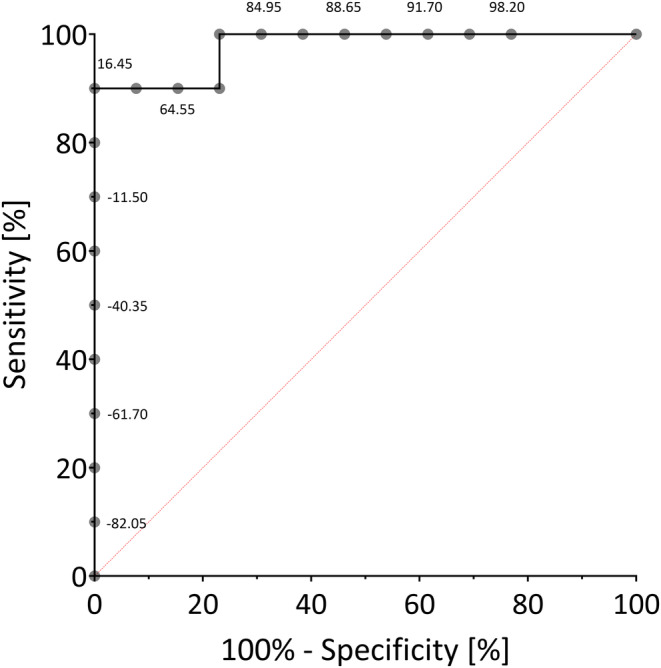
ROC curve plotting the difference “right‐sided IEDs—left‐sided IEDs” during sleep. The value pairs of sensitivity and 100% specificity for each possible cutoff are plotted and connected by the black line to form an ROC curve. Each point along the black curve corresponds to a cutoff value reflecting the extent of lateral IED dominance: Positive values indicate a right‐sided IED predominance, whereas negative values indicate left‐sided predominance. The red bisector line represents an uninformative ROC curve (sensitivity = 1 ‐specificity).

ROC curves were generated for right‐sided and left‐sided IEDs during both sleep and wakefulness. The AUC values for IEDs during sleep were .77 (*p* = .047) for right‐sided and .85 (*p* = .019) for left‐sided IEDs (Figure 2). The calculated cutoff values were 34.0% for right‐sided and 14.5% for left‐sided IEDs. For IEDs during wakefulness, the AUC values were .82 (*p* = .084) for right‐sided and .88 (*p* = .036) for left‐sided IEDs. The calculated cutoff values were 52.5% for right‐sided and 13.5% for left‐sided IEDs. As one of the AUC values of IEDs during wakefulness did not reach significance, neither was utilized for further analyses. To determine the necessary preponderance of unilateral IED_***s***_ to differentiate between left and right HHs, the difference between right‐ and left‐sided IEDs during sleep was plotted in an ROC curve (Figure 3). The resulting “difference‐ROC curve” had an AUC value of .98 (*p* = .002), with a calculated cutoff value 16.45% (sensitivity 90%, specificity 100%).

### 3.7 Binomial logistic regression

The binomial logistic regression produced a significant model shown in (A) (*p* = .004).

(A)
$$\ln\left(\frac{\hat{p}}{\left(1-\hat{p}\right)}\right)=.606+.020*\mathrm{SzO}+\left(-.008\right)*{\text{IEDs}}_{a}+.032*{\text{IEDs}}_{s}+\left(-.025\right)*\mathrm{SzS}$$

Abbreviations: IEDs_a_, side IEDs awake state; IEDs_s_, side IEDs during sleep; SzO, seizure onset side; SzS, seizure semiology side.

**FIGURE 4:**
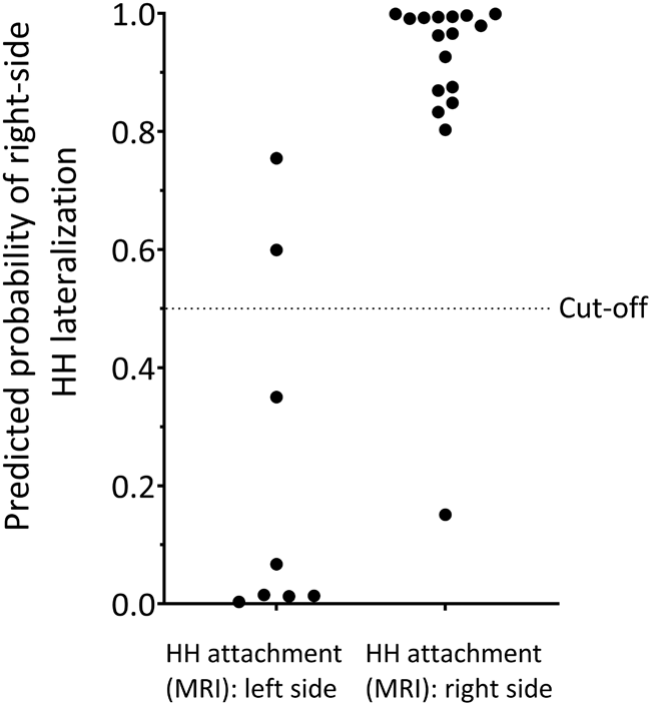
Predicted probability of accurately lateralizing HHs according to the logistic regression model.

The Akaike information criterion (AIC) for the model (A) was 24.75, superior to an intercept only model (AIC 33.52). The goodness of fit of model (A) was confirmed by the Hosmer‐Lemeshow test, with *x* ^2^ (25) =3.860 and *p* = .870, indicating a very good fit. Model (A) explained 67% of the observed variance (Tjurs *R* ^2^ = .666). The corresponding ROC curve had an AUC of .98 (95% CI .93, 1.00; *p* = .002). For predicting the approach side of the first SRT, the model (A) achieves a sensitivity of 75% and a specificity of 94% (Figure 4).

## 4 DISCUSSION

In this study, we analyzed clinical routine data from pre‐interventional VEEG of patients with HHs. As a proof of concept, we demonstrated that both ictal and interictal EEG pathologies, as well as seizure semiology, exhibit moderate to strong correlation with HH lateralization in anatomically unilateral HH, with IEDs during sleep and seizure semiology being particularly informative. In addition, in three patients with bilateral HHs, parameters correlated with the SRT‐approach side, assumed to be the functional predominant side based on sustained post‐interventional seizure freedom.

Although lateralization from electroclinical data is well‐established in other focal epilepsies^30^, ^31^ its applicability in HHs remained unclear and required further investigation. The unique intrinsic epileptogenesis and ictogenesis of a non‐cortical epileptogenic lesion even led to the statement that the utility of EEG is limited in evaluating these patients.^32^ Ictal EEG changes have been investigated extensively in various studies on focal epilepsies as parameters for lateralization, usually observed ipsilateral to the epileptogenic zone and achieving sensitivities of up to 90%.^30^, ^31^, ^33^, ^34^ Conversely, within the context of HHs, only limited research has explored the lateralization value of ictal EEG. One of the studies that contributed significantly to our understanding of the intrinsic epileptogenicity of HHs also investigated cortical changes.^35^ However, evaluation was done using multiple depth electrodes, thus introducing the potential for sampling error. Preliminary evidence from this study suggests that ictal potentials tend to cluster ipsilaterally to the HH attachment side. Another study supported the notion that the ictal onset often appears ipsilaterally to the HH attachment side but stressed that this is limited because ictal onset is often unrecognizable on scalp EEG in many seizures.^32^ In nearly all patients in this study (*N* = 21), at least one seizure onset was observed in the EEG, with 59% of all seizures classified as gelastic—the type most often lacking a discernible scalp seizure pattern. As a result, more than 50% of the cases in our study exhibited seizures without a recognizable scalp EEG pattern, and, when present, it typically appeared after the clinical onset, indicative of propagation phenomena. This underscores the challenges faced by patients with HH, for whom seizure patterns often go undetected on surface EEG due to the subcortical origin of gelastic seizures.^32^ These findings align with previous research indicating that gelastic seizures displayed no corresponding pattern in scalp EEG in 75% of cases. Consequently, ictal activity analyzed on scalp EEG may not serve as a reliable parameter for lateralizing HHs as it may reflect propagation rather than true lateralization.

Our results suggest that IEDs, particularly during sleep, are valuable lateralization parameters, lateralized predominantly ipsilateral to the HH attachment side. The phenomenon of increased IED rates during sleep is not novel and has been evidenced in various types of epilepsy.^36^, ^37^, ^38^ In addition, Conrad et al. showed that IEDs occurring during sleep tend to arise more frequently in the seizure‐onset zone, supporting our findings on the utility of IEDs during sleep for HH lateralization.^39^ However, a significant proportion of patients, especially those with bilateral HHs, show no clear IED distribution, with bilaterally synchronous IEDs being common. We determined that a predominance of 16.5% in IEDs on one side is sufficient to distinguish between left‐ and right‐attached HHs with high sensitivity and specificity despite a high rate of bilateral IEDs. Although IEDs during sleep appear valuable for HH lateralization, larger studies are necessary to validate these findings and establish both a cutoff value and a CI.

Our findings on the lateralizing value of seizure semiology demonstrate a strong inverse correlation between the attachment side of the HH and contralateral clinical semiology. It is important to note, however, that many gelastic seizures were observed without corresponding EEG changes, making it difficult to precisely determine the timing of clinical signs. As a result, the reliability of the reported lateralization signs remains uncertain. Nonetheless, the primary aim of our study was not to identify specific localization but to assess lateralization. In addition, two large published series have addressed similar questions. The first study demonstrated that an asymmetric smile during a gelastic seizure serves as a reliable lateralizing sign, exhibiting a high positive predictive value for contralateral HH attachment.^25^ Conversely, the other study failed to identify a significant correlation between unilateral motor signs (such as tonic contractions, clonic activity, and head version) and the HH attachment side.^40^ This discrepancy may stem from differences in methodology. Although the latter study analyzed whole seizures for semiologic components of known lateralizing value, potentially encompassing more propagational phenomena, our study focused solely on very early lateralizing signs. Furthermore, our logistic model revealed that the combined analysis of IEDs during sleep and semiology predicts HH lateralization with a high probability of 88%. The significance of semiology may lie in providing additional lateralization information in cases when the distribution of IEDs in the EEG is less clear.

Alternative diagnostic methods, such as SPECT coregistered to MRI (SISCOM), or 18F‐fluorodeoxyglucose–positron emission tomography (18F‐FDG‐PET), have been used by various research groups to delineate HH lateralization.^41^, ^42^, ^43^, ^44^ These techniques have been utilized primarily in a scientific context, such as in gaining understanding of subcortical networks associated with HHs, but they have significant drawbacks: high cost, resource intensity, radioactivity exposure, need for sedation, and limited availability. Particularly for pediatric patients with HHs, these diagnostic tests may not be practical for routine clinical use.

Although the post‐interventional outcome was not our study's primary focus, our findings align with those of previous research indicating that SRT is an effective minimally invasive technique for successfully disconnecting HHs from surrounding structures to achieve seizure freedom or reduction, and that early intervention is beneficial. Younger patients likely have less mature neural networks and less secondary epileptogenesis, leading to better outcomes.^11^, ^45^ Seizure freedom after one or repeated SRT was achieved in 72% of our patients, aligning with the 73.3% reported in the largest long‐term follow‐up study.^10^ Furthermore, eight patients underwent Re‐SRT for subsequent SRT, with improved outcomes observed in five cases, supporting the notion that Re‐SRT is effective when previous SRT treatments fail.^11^ This study did not specifically analyze ASM influence, as HH‐caused seizures are generally drug resistant. However, we cannot completely rule out the possibility of anti‐seizure medication (ASM) affecting outcomes. Our observations indicated that the number of ASMs administered did not appear to impact overall results.

This study has several limitations. First, as a proof‐of‐concept study, and given the rarity of HHs, particularly bilateral cases, a larger proportion of patients with unilateral HHs were examined to test the hypothesis. Although 25 patients were analyzed, and the number of seizures was large compared to previous studies, the small sample size and mixed population of unilateral and bilateral HHs restrict the strength of conclusions. The lateralizing value of IEDs and seizure semiology needs further validation in a larger cohort, particularly in the context of bilateral HHs. Expanding the sample size and including more patients with bilateral HHs would improve the external validity of the findings, ensuring that they can be applied to this rare but complex patient group.

Scalp EEG was used in this study, although it has limitations in capturing both ictal and interictal activity. Because this was a retrospective study, we relied on available data. SEEG, stereo‐electroencephalogram; a more precise diagnostic tool, could better distinguish between extra‐hypothalamic epileptogenic networks not involving the hamartoma and those simultaneously or subsequently affecting both HHs and cortex, thereby enhancing lateralization accuracy. However, SEEG is invasive, not routinely used in the pre‐interventional workup of patients with HHs, and its availability is limited. Visual EEG evaluations introduce subjectivity and inter‐rater variability.^46^ To address this, all EEG recordings were first analyzed by two independent investigators. In cases of disagreement, which occurred in <10% of events, a consensus classification was reached between the two raters in a second step. Visually unrecognized IEDs, especially in sections with low IED rates, may bias lateralization. However, as only 3 of 24 EEG studies showed fewer than 10 IEDs in 10 min of sleep, with a clear distribution favoring one side, this impact is negligible.

## 5 CONCLUSION

Our findings suggest that IEDs during sleep and lateralizing seizure semiology are effective predictors for the attachment side of unilateral HHs. We also hypothesize that these parameters may predict the functionally dominant side in bilaterally attached HHs and aid in determining the SRT approach side. However, this hypothesis requires validation in a multicenter study with a larger cohort, particularly focusing on patients with bilateral HHs.

## AUTHOR CONTRIBUTIONS

**Julia Jacobs‐LeVan:** Conceptualization (lead); investigation (supporting); writing—review and editing (equal). **Kerstin Alexandra Klotz**: Conceptualization (lead); formal analysis (lead); investigation (lead); methodology (lead); project administration (lead); resources (equal); writing—original draft (lead). **Sarah M. Metzger**: Formal analysis (supporting), investigation (equal); writing—review and editing (equal). **Friederike Niedermoser**: Conceptualization (supporting); formal analysis (lead); investigation (lead); methodology (lead); project administration (supporting); writing—original draft (lead). **Peter C. Reinacher**: Investigation (equal); resources (equal). **Jan Schönberger**: Investigation (equal); writing—review and editing (equal). **Andreas Schulze‐Bonhage**: Investigation (supporting); resources (supporting); writing—review and editing (equal). **Kathrin Wagner**: Investigation (equal); writing—review and editing (equal).

## FUNDING INFORMATION

K.A.K. and J.S. were supported by the Berta–Ottenstein–Programme for Clinician Scientists at the Medical Faculty, University of Freiburg.

## CONFLICT OF INTEREST STATEMENT

None of the authors has any conflict of interest to disclose.

## ETHICS STATEMENT

This study was approved by the ethics committee of the University Medical Center Freiburg (file 26/19). All included patients or their legal representatives gave written consent to the use of their data for research purposes. We confirm that we have read the Journal's position on issues involved in ethical publication and affirm that this report is consistent with those guidelines.

## Data Availability

The data that support the findings of this study are available from the corresponding author, upon reasonable request.
